# Treatment of children under 4 years of age with medulloblastoma and ependymoma in the HIT2000/HIT-REZ 2005 trials: Neuropsychological outcome 5 years after treatment

**DOI:** 10.1371/journal.pone.0227693

**Published:** 2020-01-23

**Authors:** Holger Ottensmeier, Paul G. Schlegel, Matthias Eyrich, Johannes E. Wolff, Björn-Ole Juhnke, Katja von Hoff, Stefanie Frahsek, Rene Schmidt, Andreas Faldum, Gudrun Fleischhack, Andre von Bueren, Carsten Friedrich, Anika Resch, Monika Warmuth-Metz, Jürgen Krauss, Rolf D. Kortmann, Udo Bode, Joachim Kühl, Stefan Rutkowski

**Affiliations:** 1 Department of Paediatric Haematology and Oncology, University Children's Hospital, University Medical Center, Wuerzburg, Germany; 2 Comprehensive Cancer Center Mainfranken, University Medical Center, Wuerzburg, Germany; 3 AbbvVie, Oncology Development, Chicago, Illinois, United States of America; 4 Department of Paediatric Haematology and Oncology, University Medical Centre, Hamburg-Eppendorf, Hamburg, Germany; 5 Institute of Biostatistics and Clinical Research, University Muenster, Muenster, Germany; 6 Pediatrics III Oncology, University Hospital of Essen, Essen, Germany; 7 Department of Paediatrics and Adolescent Medicine Division of Paediatric Haematology and Oncology, University Hospital of Geneva, Geneva, Switzerland; 8 Department of Haematology Oncology, University Children´s Hospital Rostock, Rostock, Germany; 9 Department of Neuroradiology, HIT 2000 National Reference Center, University Medical Center Wuerzburg, Wuerzburg, Germany; 10 Department of Paediatric Neurosurgery, University of Wuerzburg, University Medical Center Wuerzburg, Wuerzburg, Germany; 11 Department of Radiotherapy, University of Leipzig, Leipzig, Germany; 12 Department of Paediatric Oncology, University of Bonn, Bonn, Germany; National Cancer Institute, UNITED STATES

## Abstract

Young children with brain tumours are at high risk of developing treatment-related sequelae. We aimed to assess neuropsychological outcomes 5 years after treatment. This cross-sectional study included children under 4 years of age with medulloblastoma (MB) or ependymoma (EP) enrolled in the German brain tumour trials HIT2000 and HIT-REZ2005. Testing was performed using the validated Wuerzburg Intelligence Diagnostics (WUEP-D), which includes Kaufman-Assessment-Battery, Coloured Progressive Matrices, Visual-Motor Integration, finger tapping “Speed”, and the Continuous Performance Test. Of 104 patients in 47 centres, 72 were eligible for analyses. We assessed whether IQ was impacted by disease extent, disease location, patient age, gender, age at surgery, and treatment (chemotherapy with our without craniospinal irradiation [CSI] or local radiotherapy [LRT]). Median age at surgery was 2.3 years. Testing was performed at a median of 4.9 years after surgery. Patients with infratentorial EPs (treated with LRT) scored highest in fluid intelligence (CPM 100.9±16.9, mean±SD); second best scores were achieved by patients with MB without metastasis treated with chemotherapy alone (CPM 93.9±13.2), followed by patients with supratentorial EPs treated with LRT. In contrast, lowest scores were achieved by patients that received chemotherapy and CSI, which included children with metastasised MB and those with relapsed MB M0 (CPM 71.7±8.0 and 73.2±21.8, respectively). Fine motor skills were reduced in all groups. Multivariable analysis revealed that type of treatment had an impact on IQ, but essentially not age at surgery, time since surgery or gender. Our results confirm previous reports on the detrimental effects of CSI in a larger cohort of children. Comparable IQ scores in children with MB treated only with chemotherapy and in children with EP suggest that this treatment strategy represents an attractive option for children who have a high chance to avoid application of CSI. Longitudinal follow-up examinations are warranted to assess long-term neuropsychological outcomes.

## Introduction/Background

Therapy for malignant brain tumours aims to improve survival rates and minimise long-term sequelae. In young children, this represents an extremely challenging task, because some brain tumours in this age group have a particularly aggressive biology and the younger brain is more susceptible to therapy-induced damage. Thus, in the German paediatric brain tumour group, children with medulloblastoma (MB) and ependymoma (EP) younger than 4 years of age were stratified into different risk-adapted treatment regimens [1–3].

Neuropsychological outcome data are important to weigh the benefits of better survival with more intense therapy against the risks of long-term sequelae [4]. Initial neuropsychological follow-up examinations were conducted with a limited number of patients (n = 34) in the HIT-SKK’87/’92 cohorts. Those examinations showed a significant decline in fluid intelligence after craniospinal irradiation (CSI) treatments and a significantly better outcome in children treated only with chemotherapy including intraventricular methotrexate (MTX^i.vt.^) instead of CSI [2, 4–8]. However, it is well established that the neurocognitive outcome can also be influenced by other factors, such as the type and location of the tumour [9–13], the age at the time of treatment [5, 14–16], the type and dose of radiotherapy [5, 6, 15, 17, 18], and the type of chemotherapy [1, 19]. Although chemotherapy alone generally causes less severe late cognitive effects than CSI, it may nevertheless have an impact on neurocognitive functions, such as attention, executive functioning, visual processing, and visual-motor functioning. A close correlation has been established between the total IQ score and visual-motor and executive functions [20]. In addition, other factors might impact intellectual development, such as tumour histopathology, hydrocephalus and its management, or postoperative posterior fossa syndrome (PFS) [21]. A recent review described impairments in 456 survivors of childhood posterior fossa tumours and concluded that MB survivors exhibited substantial restrictions in IQ, attention, as well as executive and memory functions [21]. In contrast, von Hoff et al. reported a surprisingly good neuropsychological outcome in EP survivors; only 2 of 23 patients had impaired IQs [3].

At the time of initiation of the HIT-2000 trial, there was a striking lack of standardization in conducting neuropsychological follow-up examinations, based on functional domains and/or a taxonomic framework [22, 23]. Therefore, we developed and extensively validated two test batteries: the “Wuerzburger Psychologische Diagnostik” (WUEPD) and the Wuerzburg short diagnostic (WUEP-KD), first presented at the ISPNO 2000 [24]. Our group has recently published the validity and reliability of these test batteries for use in children with brain tumours [7].

The present cross-sectional study aimed to confirm the detrimental effects of CSI in a larger cohort of MB and EP patients, recruited from the HIT-2000 studies, 5 years after surgery. We also aimed to analyse the impact of therapy-related factors (i.e., local radiotherapy [LRT], CSI, MTX^i.vt.^) as well as age, gender, or time since surgery on the IQ profiles of patients by comparing outcomes in the prospective HIT2000/HIT-Rez 2005 trials. Here, we report on children treated before the HIT-2000 trial amendment in 2005, and who were examined between 2007 to 2011.

## Subjects and methods

### Psychological tests

All tests were applied previously for the diagnosis of treatment-related side effects in paediatric patients with brain tumours in German-speaking countries; the tests are listed in Table 1. As a basis, we chose the Cattell-Horn-Carroll (CHC) model of intelligence [7, 24, 25]. Our model-oriented tests were performed with the WUEPD [7] full test battery, which requires about 3 h of evaluation with the patient. The WUEPD assessment of intelligence consisted of the WUEP-KD, a theory-oriented diagnostic tool for neuropsychological follow-up, and the Kaufman Assessment Battery for Children (K-ABC) [7, 23, 26]. At the time of study initiation (1996–2000), only the K-ABC and the HAWIK-R were available in a german, standardised version of 1991, however, the latter did not meet the CHC-based requirements.

**Table 1:** Neuropsychological tests and abbreviations. All standardised scores (response variables) were normalised to mean 100, SD 15.

**Neuropsychological Score**	**Psychological Test Battery WUEPD**
Short Test	Mental Intelligence Scores (WUEP-KD)
CPM	Coloured Progressive Matrices
VMI	Developmental Test of Visual-Motor Integration
K_NR	Kaufman-Assessment Battery for Children, “Number Recall”
K_RI	Kaufman-Assessment Battery for Children, “Riddles”
**IQ Test Battery**	**Kaufman Assessment Battery for Children**
K_MPC	K-ABC Mental Processing Composite
K_SIM	K-ABC Simultaneous Processing
K_SEQ	K-ABC Sequential Processing
**Psychomotor Functions**	**Psychomotor Abilities (Speed Tapping Test)**
T_SP	Tapping Speed
**Executive Functions**	**Executive Functions**
CPT-k_F	Continuous Performance Test: Hits/false
CPT-k_DT	Continuous Performance Test: Selective Decision Speed
CPT-k_PO	Continuous Performance Test: Power
**Participation**	**Involvement in Daily Life Situation**
FMH Questionnaire	Performance scale, Fertigkeitenskalen Münster-Heidelberg (FMH)

Standard tests commonly lack several important features: they do not differentiate between specific neurocognitive aspects of IQ; they do not discriminate between affected brain areas; and they do not consider brain damage. To provide information about the performance of specific brain regions, the applied subtests must at least distinguish between frontal, cranial, and cerebellar parts of the brain. Importantly, the WUEP-KD mental test battery comprised tests that could detect brain damage independent of the motor response abilities of the tested patients.

To evaluate the time needed to achieve a positive motor response, we examined two variables with computerised tests, the cerebellar time-modulated motor-oriented tap-time (or tapping speed, TS) and the fronto-cortical oriented processing speed (CPT-k with the subtests hits false, decision time, and power, -F, -DT-, -PO). Because these tests were measured separately from mental tests, they avoided the bias of impaired motor function, which might have influenced the results [27, 28].

To assess the degree of impairment in behaviour and participation in daily life situations we employed the “Assessment Scales of Involvement in Life Situations” (Fertigkeitenskala Münster Heidelberg; FMH) [29].

General intelligence was measured with the K-ABC, which included the total IQ Mental Processing Composite (K_MPC) and the subtests Simultaneous Processing (K_SIM) and Sequential Processing (K_SEQ, Table 1). Central cognitive ability was dertermined as an overall ‘fluid intelligence’ score from the Raven Coloured Progressive Matrices [30] with the standardization of Bulheller & Häcker in 1218 children. This test takes the fundamental cerebral networks into account [6, 31].

To detect disturbances in the appropriation of environmentally oriented features, we used the developmental test of Visual-Motor Integration (VMI) [32]. According to the theories of Luria [33] and Piaget [34], in younger children, shape detection is the best method for analysing feature detection. K.E. Beery showed that the ability of reproducing a shape is part of a complex visuomotoric integration process according to Piaget, and is therefore a self-generated performance of intelligence of the child [35].

For testing verbal functions and the active vocabulary, we applied the Riddles of the K-ABC (K_RI) test. This test explores comprehension-knowledge (Gc) [23]. Short-term working memory (Gsm) was measured with the subtest “Number Recall” (KABC-NR) [26]. To test motoric functions we used a computerised morse key which is able to measure two capabilities separately: First, the higher order domain of cognitive processing speed was measured with a short version of the Connors Continuous Performance Test (CPT-k) for selective attention [36]. Furthermore, we introduced a combined parameter, decision stability or power (CPT-k_PO), which was calculated as the mean of the false rate CPT-k_F and CPT-k_DT. Second, fine motor dexterity was assessed by measuring finger tapping performance with the parameter Tapping Speed (T-SP) [37]. The retest reliability scores for all applied tests were derived from either published manuals or our own studies [7, 38].

### Patients

Between January 2007 and April 2011, 104 patients under 4 years of age underwent neuropsychological follow-up examinations. Eligibility criteria for inclusion into the test programme was a history of surgery for a brain tumour and subsequent treatment, according to the procedures specified in the multi-centre HIT2000 and HIT-REZ2005 trials. The clinical trial was reviewed and approved by the IRB of the University of Würzburg and the competent authorities. All participants or their guardians gave their informed consent. Although some patients were lost to follow-up, the vast majority of patients in the two trials were stringently tested in a standardised way; thus, the results can be considered representative of the entire HIT2000/HIT-REZ2005 study population. The patients underwent a neuropsychological examination at a median of 4.9 years after first surgery [1, 2]. To minimise fluctuations due to inter-rater reliability, all examinations were carried out in the respective centres on site by one trained neuropsychologist. Of the 104 tested patients, 23 and 9 patients had to be excluded due to treatment deviations from the protocol or due to divergent histology. Thus, 72 patients with MB and EP were included in the final analysis. Details on patients, the different treatment arms, including the intended and actually applied radiation dosages are detailed in Table 2.

**Table 2:** Treatment groups of medulloblastoma and ependymoma in children < 4 of age.

Study groups	Group characteristics	Chemotherapy (CT)	Radiotherapy	Study Arm
**MBP** (P for polychemotherapy)	primary medulloblastoma without metastasis	SKK CT + intraventricular MTX	none	HIT2000-BIS4
**MBR** (R for irradidation)	relapsed medulloblastoma without metastasis	Systemic CT + intraventricular MTX + HDCT	per protocol: 24 Gy to brain/spine in five weekly fractions of 1.6 Gy followed by boost to the posterior fossa to 54 Gy in daily fractions of 1.8 Gy. mean applied dose: 54 Gy	HIT-REZ-2005
**MBRM** (RM for radiation with metastasis)	primary medulloblastoma with metastasis	CARBO/ETO-96h CT + intraventricular MTX +/- HDCT	per protocol: 24 Gy to brain/spine in five weekly fractions of 1.6 Gy followed by boost to the posterior fossa to 54,6 Gy in daily fractions of 1.8 Gy. mean applied dose: 54 Gy	MET-HIT2000-BIS4
**EPI** (I for infratentorial location)	Infratentorial ependymoma	SKK CT	per protocol: 54 Gy focal radiation therapy to the tumor bed with 2 cm safety margin, five weekly fractions of 1.8 Gy mean applied dose: 53 Gy	E-HIT2000-BIS4
**EPS** (S for infratentorial location)	supratentorial ependymoma	SKK CT	per protocol: 54 Gy focal radiation therapy to the tumor bed with 2 cm safety margin, five weekly fractions of 1.8 Gy mean applied dose: 51 Gy	E-HIT2000-BIS4

### Treatments

Children with localised MB received systemic chemotherapy and MTX^i.vt.^ to avoid CSI. Until the 2005 amendment, all patients with MB were treated primarily with SKK chemotherapy, and received CSI only when there were metastases or in case of a non-response to chemotherapy. MB patients with metastasis were treated with combinations of induction chemotherapy, response-adapted high-dose chemotherapy and CSI. Children with EP received the HIT-SKK chemotherapy, but without MTX^i.vt.^, followed by local radiotherapy (LRT) [39, 40]. For this neuropsychologcial outcome study, children with MB were categorised into three groups: primary disease without metastases that received polychemotherapy, including MTX^i.vt.^ (MBP, n = 19), relapsed without metastases that received polychemotherapy, including MTX^i.vt.^ and CSI (MBR, n = 5), and primary disease with metastases that received MTX^i.vt.^ and CSI (MBRM, n = 6). Those with EP were categorised as: infratentorial (EPI, n = 32; n = 5 grade II, n = 27 grade III) and supratentorial (EPS, n = 10; all grade III).

### Statistical analysis

Individual age-corrected test scores are standard scores (SS), synonymous to IQ. This mean, non-age-related IQ-score is 100 with a single standard deviation (SD) of 15. All applied tests are listed in Table 1.

Univariable distributions of metric variables are described by mean and SD, when data are normally distributed. Otherwise, they are described by median and range. For categorical variables, absolute frequencies are stated. Associations between a normally distributed metric outcome and nominal predictor variables were assessed with the t-test or analysis of variance (ANOVA), depending on whether two or more independent samples were compared.

For multivariable analyses, linear fixed effect models were applied using a stepwise variable selection procedure recommended by Collett [41] to analyse the simultaneous impact of the variables age at surgery (continuous: years), time from start of treatment to neuropsychological testing (TtoNT, continuous: years), gender (binary), and treatment group (nominal: MBP, MBR, MBRM, EPI, EPS). This allows to estimate adjusted mean neuropsychological scores (by treatment group), adjusted for potential heterogeneity in the distribution of age at surgery, gender and TtoNT. Missing values were treated as missing at random (MAR).

Analyses were performed with the SPSS software package (version 24; IBM Inc., Armonk, NY, USA). All analyses were considered as exploratory and p-values were interpreted descriptively.

## Results

### Univariable analysis

**Fig 1:**
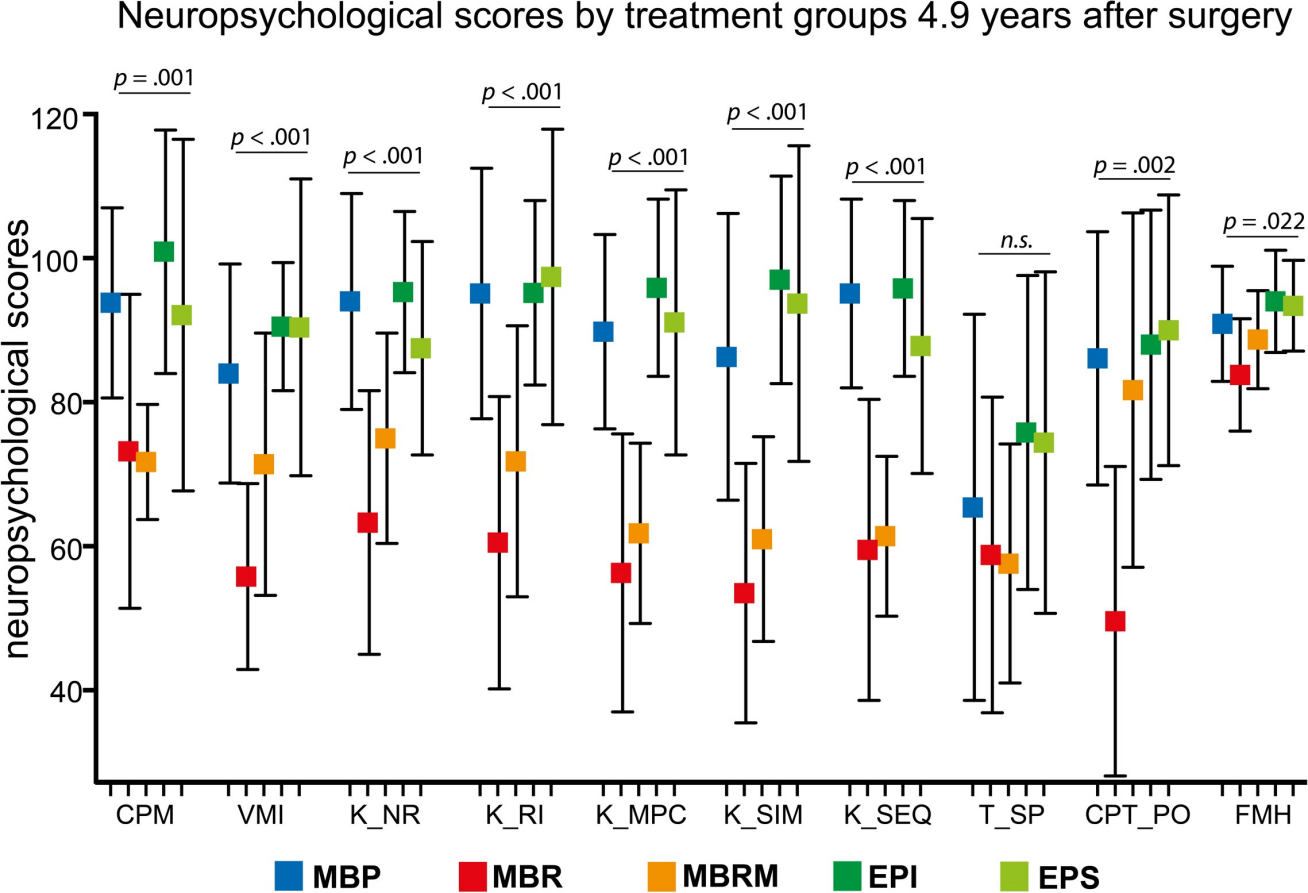
Neuropsychological outcome of children with MB and EP 4.9 years after surgery. Test results of ten different neuropsychological tests in the five different treatment groups are shown. Results are displayed as means ± standard deviation.

The median age of the 72 patients (48 males, 24 females) included was 2.3 years (range 0.6–3.8 years) at first surgery. The median age at neuropsychological testing was 7.5 years (range 4.5–12.0 years) and the median time from surgery to testing 4.9 (3.6–8.6). Fig 1 visualises results of 10 of the applied test batteries in the five treatment cohorts. Standard deviations were generally high indicating substantial variability in the different scores. In view of the small sample size, the EP subgroups (EPI, EPS) were not further divided into groups of patients with and without relapse.

For the majority of neuropsychological scores (CPM, VMI, K-NR, K-RI, K-MPC, K-SIM, K-SEQ, T-SP, CPT-k_PO, and FMH) ANOVA suggested that treatment had statistically noticeable impact on scores (Fig 1). EP subgroups generally had higher scores than MB patients. Within the MB subgroups, patients without CSI (MBP) exhibited better performance than patients receiving CSI (MBR and MBRM). Notably, tapping speed and the executive function ‘decision time’ was reduced in all groups without a relevant difference between the groups, irrespective of whether CSI was applied or not (Fig 1).

### Multivariable analysis

Multivariable analysis revealed that gender and TtoNT had no relevant impact on any of the neuropsychological outcome scores (data not shown); i.e. these variables were not selected for the final model. As expected from univariable testing, treatment modalities had a highly noticeable impact on all scores from the WUEP-KD, K-ABC, CPT-k PO, and FMH (Table 4). For the scores from the WUEP-KD and the K-ABC, treatment modality was the only selected prognostic factor (Table 3, i.e. the multivariable model reproduces the univariable analysis). In contrast, outcomes in executive capabilites (CPT-k PO) and participation in daily life (FMH) were effected by both age at surgery and treatment modality; i.e. age at surgery and treatment modality both remained as parameters for model calculations of predicted scores (Table 3).

**Table 3:** Results of multivariable model building, summarised over all neuropsychological scores. For each neuropsychological score, the multivariable model building assessed the simultaneous prognostic impact of the variables treatment group (nominal: MBP, MBR, MBRM, EPI, EPS) and age at surgery (continuous: years). P-values indicate whether the potential explanatory variable was identified as independent prognostic factor in the multivariable model. Not selected variables were indicated by “N/S”.

NP Test	Available cases	Treatment Groups	Age at first surgery
	**Main Mental Intelligence Scores (WUEP-KD)**		
**CPM**	72	0.001	N/S
**VMI**	72	< 0.001	N/S
**K-NR**	72	< 0.001	N/S
**K-RI**	70	< 0.001	N/S
**Kaufman Assessment Battery for Children (K-ABC)**			
**K-MPC**	72	< 0.001	N/S
**K-SEQ**	72	< 0.001	N/S
**K-SIM**	72	< 0.001	N/S
**Psychomotor Abilities I: Fine Motor Dexterity (Tapping Speed)**			
**T-SP**	72	N/S	N/S
**Psychomotor Abilities II: Executive Functions (CPT-k)**			
**CPT-k_F**	56	< 0.001	< 0.001
**CPT-k_DT**	72	N/S	N/S
**CPT-k_PO**	55	0.001	0.014
**Participation(FMH)**			
**FMH**	69	0.008	0.009

**Table 4:** Multivariable linear model for WUEP-KD score at the 5-year follow-up for the complete cohort of patients. Bold indicates statistically noticeable estimated differences. * Wald test. ** Predicted means adjusted for mean age at surgery.

Treatment	Group	Predicted Mean	95% CI	Difference from MBP	P*	Predicted mean	95% CI	Difference from MBP	P*
		**CPM**				**K-ABC Mental Processing Composite**			
**MTX** ^**I.VT.**^	**MBP** (n = 19)	93.8	85.9, 101.7	-	-	89.8	83.3, 96.3	-	-
**MTX** ^**I.VT.**^ **and CSI**	**MBR** (n = 6)	73.2	59.1, 87.2	**-20.6**	**0.013**	56.3	44.8, 67.9	**-33.5**	**<0.001**
	**MBRM** (n = 5)	71.7	56.3, 87.1	**-22.1**	**0.013**	61.8	49.1, 74.5	**-28.0**	**>0.001**
**Local RT**	**EPI** (n = 32)	100.9	94.8, 107.0	7.1	0.158	95.9	90.9, 100.9	6.1	0.145
	**EPS** (n = 10)	92.1	81.2, 103.0	-1.7	0.803	91.1	82.1, 100.1	1.3	0.821
		**VMI**				**K-ABC Sequential Processing**			
**MTX**	**MBP** (n = 19)	84.0	77.7, 90.2	-	-	95.1	86.7, 101.5	-	-
**MTX** ^**I.VT.**^ **and CSI**.	**MBR** (n = 6)	55.8	44.6, 67.0	**-28.1**	**<0.001**	59.5	48.1, 70.9	**-35.6**	**<0.001**
	**MBRM** (n = 5)	71.4	59.1, 83.7	-12.5	0.073	61.4	48.9, 73.9	**-33.7**	**<0.001**
**Local RT**	**EPI** (n = 32)	90.5	85.7, 95.3	6.6	0.102	95.8	90.8, 100.7	0.6	0.874
	**EPS** (n = 10)	90.4	81.7, 99.1	6.5	0.233	87.8	79.0, 96.6	-7.3	0.187
		**K-ABC Number Recall**				**K-ABC Simultaneous Processing**			
**MTX** ^**I.VT.**^	**MBP** (n = 19)	93.9	87.7, 100.2	-	-	86.3	78.3, 94.2	-	-
**MTX** ^**I.VT.**^ **and CSI**	**MBR** (n = 6)	63.3	52.2, 74.4	**-30.6**	**<0.001**	53.5	39.3, 67.7	**-32.8**	**<0.001**
	**MBRM** (n = 5)	75.0	62.8, 87.2	**-18.9**	**0.007**	61.0	45.4, 76.5	**-25.2**	**0.005**
**Local RT**	**EPI** (n = 32)	95.3	90.5, 100.1	1.4	0.730	97.0	90.9, 103.2	**10.7**	**0.037**
	**EPS** (n = 10)	87.5	78.9, 96.1	-6.4	0.230	93.7	82.7, 104.7	7.4	0.277
		Continuous Performance Test–k Power**				**Assessment Scales of Involvement in Daily Life (FMH)**			
**MTX** ^**I.VT.**^	**MBP** (n = 18)	87.2	78.7, 95.7	-	-	90.9	87.6, 94.3	-	-
**MTX** ^**I.VT.**^ **and CSI**	**MBR** (n = 6)	48.8	33.3, 64.4	**-38.4**	**<0.001**	83.6	78.0, 89.3	**-7.3**	**0.029**
	**MBRM** (n = 5)	74.4	53.5, 95.3	-12.8	0.263	87.2	81.0, 93.5	-3.7	0.301
**Local RT**	**EPI** (n = 31)	88.2	80.9, 95.4	1.0	0.86	94.3	91.8, 96.8	3.3	0.117
	**EPS** (n = 10)	90.2	77.0, 103.3	3.0	0.705	93.2	88.8, 97.5	2.2	0.426

The predicted mean scores of the various neuropsychological tests calculated in the final model are given in Table 4 along with the estimated differences between the MBP (reference score) group and the other four treatment groups: children with EPI always achieved highest scores followed by children with either EPS or MBP. All patient groups treated with CSI performed substantially worse than children treated without CSI. This pattern was also sustained for participation in daily life situations (FMH questionnaire). These data confirm that the different treatment modalities remain the prime prognostic factors for neuropsychological outcome. Compared to that other factors such as age at surgery are only of subordinate significance for IQ development of childhood brain tumor survivors.

## Discussion

In the present study, we assessed the neuropsychological outcome of 72 consecutive children with MB or EP that were treated with protocols specified in the HIT2000 and HIT-REZ-2005 trials. Although this cohort represented one of the largest groups of children analysed with a uniform test battery to date, the numbers of patients included in the subgroups were still relatively small. Multivariable analysis revealed that age at surgery, gender, and TtoNT had essentially no relevant impact on the test scores. In order to minimise the influence of unequal testing intervals we only included children tested 5 years after diagnosis into the analysis.

The multivariable analysis confirmed that both groups treated with CSI (MBR and MBRM) displayed inferior intellectual functions compared to those treated without CSI. Analyses revealed a difference in the predicted means of up to 35 IQ points between the children treated only with chemotherapy including MTX^i.vt.^ and those that received additional CSI. Furthermore, these data show that a deferral of CSI beyond the age of 18 months was not sufficient to prevent cranial radiation-induced injuries to the CNS [42].

The devastating effect of irradiation on the developing brain of young children was initially demonstrated in the HIT’87/’92 studies. Those data also demonstrated that chemotherapy including MTX^i.vt.^ was significantly less harmful than CSI [2, 7]. CPM test results in our study confirmed the detrimental impact of CSI on fluid intelligence. Recent MRI data support these findings by showing areas of reduced cortical thickness after MB treatment (including CSI) and a link between intellectual performance and the right prefrontal white matter volume [31, 43]. As reported before, CSI also significantly reduced working memory (K_NR) in surviving children. In contrast to irradiation, chemotherapy and MTX^i.vt.^ alone appeared to be less toxic. IQ scores in children receiving chemotherapy and MTX^i.vt.^ without CSI (group MBP) remained within normal age-adjusted limits for standard score results [2, 44]. Importantly, the clinical noninferiority of MTX^i.vt.^ as a substitute for CSI has also been demonstrated, at least in young patients with MB that displayed a desmoplastic or extensive nodularity histology, confirming that irradiation is dispensable for children with low-risk MB [1]. As a logical development, the 2005 amendment attempted to prevent CSI for a greater number of patients with metastasised MB, implementing an intensified induction chemotherapy to improve initial tumour control followed by high-dose chemotherapy instead of CSI for consolidation. In summary, our data consistently show that CSI represented the key risk factor for intellectual damage in young children with MB [1, 44–47]. Whether upfront MTX^i.vt.^ contributes to CSI toxicitiy remains to be investigated in future trials or subgroup analyses.

Furthermore, it is conceivable that other factors, such as hydrocephalus or posterior fossa sydrome (PFS), might also have impacted intellectual development. Unfortunately, hydrocephalus and its treatment was not recorded as a separate risk factor in the respective trials. In our cohort, we could detect only minor speaking abnormalities, such as low voice, slowed speech, and articulation weaknesses, in 16 patients with postoperative symptoms of PFS 5 years after surgery. However, to date, PFS has been an ill-defined entity; therefore, these data warrant confirmation in trials with a specialised design.

A comparison of the two EP cohorts offered further important insights. Previously, we reported overall IQ scores within the normal range for 23 children with EP treated with LRT [3]. Based on the present study results, we could further differentiate between groups of children with different tumour locations. In fluid intelligence, patients with supra- as well as infratentorial EPs had the same outcome as patients with MB treated without CSI. Similarly, both EP groups showed surprisingly good results on the K-ABC cognitive domain scores and the more central cognitive WUEP-KD scores. Only speed tapping scores were below average in all groups, including both EP groups, indicating that tumour location alone is not a predictor of motor deficits. Deficits in tapping scores are particularly suited to demonstrate detrimental effects of the tumour and its treatment on cerebellar time modulation. In contrast to tapping speed, cognitive motor decisions (CPT_PO) were only delayed in patients receiving CSI (groups MBR and MBRM), showing that motor and psychomotor processing speed is differentially affected by brain tumor treatment.

Although EP tumour location did not seem to play a pivotal role in the neuropsychological outcome in our cohort, IQ scores tended to be lower in the EPS group than in the EPI group, possibly due to the closer anatomic proximity of the tumour to the frontal lobe and cortical regions. The precise anatomic tumour location was not considered an independent variable in our cohort. Perhaps the most relevant new finding from our study is that patients with MB treated with chemotherapy and MTX^i.vt.^ alone displayed equivalent or almost equivalent IQ scores than both EP groups treated with LRT without MTX^i.vt.^ on all tests that were not related to motor functioning (differences were 4.4–13.5 IQ points). This observation might partly be explained by divergent tumour genetics or different anatomic locations. However, it indicates that irradiation limited to the involved tumour field appears to be far less toxic than CSI, and that the HIT-SKK chemotherapy with MTX^i.th.^ is an acceptable option e.g. for young children with non-metastatic desmoplastic MB, both in terms of survival and long term IQ development. Data from the FMH questionnaire yielded results very similar to those from the WUEPD and WUEP-KD IQ batteries. The results illustrate the relevance of IQ for daily life situations. Therefore, the more feasible FMH questionnaire might be equally suited to identifying affected populations, compared to the more time consuming test batteries, albeit in less detail. For example, the FMH questionnaire cannot provide a sophisticated profile that can inform the design of deficit-specific rehabilitation programmes. For these purposes, the more in-depth analysis profile of the aforementioned test batteries would be required. This distinction is particularly important in examining young children, where a wide range of cognitive abilities must be investigated [48], due to the greater prevalence of neuropsychological deficits in the young age group compared to older children. Previous studies in young patients with brain tumours mainly used arbitrarily selected tests, which were not based on a precise model, like the CHC [49]. However, our data clearly showed that it is of prime importance to implement theory-driven neuropsychological test systems in this vulnerable patient population [23]. In this respect, a recently established consensus between 18 participating European countries for an internationally accepted test battery for follow-up of childhood ependymoma survivors, that can also be used for other brain tumors including medulloblastoma, represents a big leap forward. The ‘Core-Plus’ concept aims to establish a minimum dataset where resources are limited, whilst maintaining scope for a more comprehensive assessment where feasible. The model represents a significant improvement in the ability for international collaboration using the same analogous measures, which in the core battery relies upon the Wechsler IQ test to obtain IQ estimate, Verbal, Working Memory and Processing Speed. This core battery is combined with a reading subtest, a pegboard test to assess fine motor functioning, tapping speed and the CPT, the Beery VMI and Ravens Matrices, all tests that were already used in this current study. Furthermore, the additional tests include gold standard measures of executive functioning, memory, attention and academics, which are extremely important domains to clearly elucidate the late effects of paediatric brain tumor treatment, that will also drive the focus of future interventions.

## Conclusions

The WUEPD/WUEP-KD tests proved to be a reliable tool for measuring the cognitive outcome of paediatric patients with brain tumours, and were the starting point of the recently proposed CorePlus consensus concept of the *European Paediatric Brain Tumour Group* for assessment of survivors of childhood brain tumours over five years of age [7, 49].

Our data identify type of treatment as the most relevant independent risk factor for neuropsychological outcomes in young children under 4. Children receiving CSI and tested 4.9 years after surgery displayed severe limitations in key areas of cognitive development, motor function and selective attention. With respect to motoric functions, all patients displayed significant deficits in motor speed, however, reduction of cognitive motor decision time was only prevalent in children receiving CSI. Importantly, IQ scores of MB children treated with MBP (including MTX^i.vt.^) reached almost equivalent to IQ-scores of children with EP, suggesting that MBP treatment is an interesting option for children with low-risk MB who have a high chance to avoid the application of CSI. Due to the multitude of possible influencing factors, the limited number of patients in our cohort, and the clinical trial design, we could not consider all relevant parameters. Future studies are needed to determine the impact of the exact anatomic tumour location, of intrathecal chemotherapy on subsequent radiotherapy, and surgical complications on neuropsychological outcome. Longitudinal studies that focus more on IQ development over time are currently under way.

## Abbreviation

ANOVA: Analysis of Variance
CHC: Cattell-Horn-Carroll theory of intelligence
CNS: Central Nervous System
CPT-k: Continuous Performance Test
CPT-F: CPT-k Test, Score: False
CPT-DS: CPT-k Test, Score: Decision Speed
CPT-Po: CPT-k Test, Score: Power
CPM: Coloured Progressive Matrices Test
CSI: Craniospinal Irradiation
Δ: Mathematical range: delta
EP: Ependymoma
EPI: Ependymoma, infratentorial location
EPS: Ependymoma, supratentorial location
FMH: Questionnaire, Performance Scale (Fertigkeitenskala Münster Heidelberg)
G: CHC theory: general Intelligence
Gc: CHC theory: crystallised Intelligence
Gps: CHC theory: psychomotor Speed
Gsm: CHC theory: short-term Memory
Gv: CHC-theory: visual-spatial Processing
HIT2000: German brain study 2000 for infants (Hirntumor 2000)
HIT-Rez 2005: German brain study 2005 for infants with relapses
HIT-SKK´87/´92: German pilot brain study 1987/1992 for Infants (Hirntumor 1987/92)
IQ/SS: Intelligence Quotient, synonymous to Standard Score
ISPNO 2000: International Symposium on Paediatric Neuro-Oncology 2000
K-ABC: Kaufman Assessment Battery for Children
K-ABC-NR: K-ABC subtest: Number Recall
K-ABC-RI: K-ABC subtest: Riddles
K-ABC-SEQ: K-ABC subtest: Sequential Processing
K-ABC-SIM: K-ABC subtest: Simultaneous Processing
K-MPC K-ABC: all intelligence subtest groups: Mental Processing Composite
LRT: Local Radiotherapy
MAR: Missing at random
MB: Medulloblastoma
MBP: primary medulloblastoma group A, no metastases, treatment with polychemotherapy but no CSI
MBR: relapsed medulloblastoma without metastases, with CSI
MBRM: primary medulloblastoma with metastasis, with CSI
MTX^i.vt.^: Methotrexate intraventricular
PFS: Posterior Fossa Syndrome
PNET: Primitive Neuroectodermal Tumour
SPSS: Statistical Package for Social Sciences (SPSS 22), IBM
SPSS: TtoNT SPSS Test of Treatment to Neuropsychological Tests
T-SP: Tapping Score: Speed
UNIANOVA: SPSS Univariate ANOVA
VMI: Developmental Test of Visual-Motor Integration
WUEPD: Wuerzburg Intelligence Diagnostics
WUEP-KD: Wuerzburg Intelligence Short Diagnostics
WUEP-UKD: Wuerzburg Intelligence Ultra-Short Diagnostics
